# Isolated cerebral aspergillosis in an immunocompetent woman on treatment for bacterial infected necrotizing pancreatitis: A case report

**DOI:** 10.1097/MD.0000000000008908

**Published:** 2017-12-01

**Authors:** Shaoyang Zhang, Qinghui Fu, Qi Chen, Ting-bo Liang

**Affiliations:** a Department of SICU; b The Department of Hepatobiliary and Pancreatic Surgery, the Second Affiliated Hospital, Zhejiang University School of Medicine, Zhejiang Province, China.

**Keywords:** cerebral aspergillosis, immunocompetence, infected necrotizing pancreatitis, sepsis

## Abstract

**Rationale:**

Cerebral aspergillosis (CA) is a rare manifestation of invasive aspergillosis. It usually affects seriously immunocompromised hosts. Pancreatic bacterial or/and fungal infection is common in patients with severe acute pancreatitis.

**Patient concerns:**

We report the first case of an immunocompetent woman with infected necrotizing pancreatitis due to multidrug resistant *Acinetobacter baumannii* who, in the course of treatment, developed isolated CA.

**Diagnoses:**

Magnetic resonance imaging, rather than computed tomography, revealed latent homolateral sinus disease—the possible source of the Aspergillus infection.

**Interventions:**

The pancreatic infection was controlled by open necrosectomy, and the CA was disappeared after neuronavigation-guided drainage and voriconazole antifungal therapy.

**Outcome:**

The patient was discharged without complications. Our report revealed that persistent hyperglycemia, sepsisassociated immunoparalysis, and prolonged antibiotic use could impair severe patient's immunocompetence, making them more susceptible to opportunistic cerebral Aspergillus infection; the risk may be especially high in patients with paranasal sinus diseases.

**Lessons:**

Timely neurosurgical intervention combined with voriconazole antifungal therapy can provide a favorable outcome

## 1 Introduction

Cerebral aspergillosis (CA) is an opportunistic fungal infection that usually affects seriously immunocompromised hosts, typically patients on cytotoxic chemotherapy or immunosuppressive therapy, those receiving long-term corticosteroids, or those with neutropenia or immunodeficient states such as AIDS.^[1]^ However, there is an increasing number of reports of CA in immunocompetent patients, especially in the intensive care unit (ICU).^[2–4]^ Without effective management, CA has a poor prognosis. Often, the outcome depends on the immune status of the host. The mortality rate is 10% to 20% in immunocompetent patients without systemic involvement versus 90% to 100% in immunocompromised patients.^[2,3]^

Pancreatic bacterial or/and fungal infection is common in patients with severe acute pancreatitis. We describe a previously immunocompetent woman with infected necrotizing pancreatitis (INP) due to multidrug resistant *Acinetobacter baumannii* who, in the course of treatment, developed CA. To our knowledge this is the first report of CA in patients with pancreatitis. The possible causes and treatment of CA following INP are discussed.

## 2 Case report

A 72-year-old female with history of hypertension and secondary hypothyroidism was admitted to a regional hospital for an attack of acute pancreatitis. She had no other disease and no apparent immune deficiency. On the third day, she developed circulatory collapse with acute respiratory distress syndrome. She was transferred to the ICU, intubated, and started on mechanical ventilation. Supportive therapy with fluid resuscitation, pain relievers, oxygen administration, and antiemetics was provided as necessary. The circulatory failure responded to vasopressors and fluid resuscitation and was stabilized after 3 days. However, she developed high-grade fever, and empirical antibiotic therapy was started (first, with ceftriaxone and then a carbapenem). Small doses of corticosteroids were also administered intermittently to control her hyperpyrexia, but the fever persisted. Her blood glucose levels were also persistently high (>10 mmol/L). As she had severe abdominal distention, she was on total parenteral nutrition. On day 18, because of persistent severe abdominal distention and hyperpyrexia, she was transferred to our institution.

At our hospital, symptomatic treatment and close monitoring were continued. Partial enteral nutrition was started. Because of persistent systemic inflammatory response syndrome (SIRS) and suspected of sepsis, broad-spectrum antibiotics were continued (initially imipenem, with vancomycin added later). The hyperglycemia was reduced with insulin, but there were wide swings in blood sugar levels. On day 22, contrast-enhanced computed tomography (CECT) revealed extensive parenchymal necrosis of the body and head regions of the pancreas. Despite full doses of broad-spectrum antibiotics, the patient continued to spike fevers. Infected pancreatic necrosis was suspected and percutaneous catheter drainage of the necrotic collections was performed on day 27. Cultures of peripancreatic drainage fluid grew multidrug resistant *A baumannii*, and antibiotic treatment with tigecycline and imipenem was started. Since she showed no response, open necrosectomy was done on day 31. Tigecycline and imipenem were administered postoperatively for 2 weeks, after which tigecycline was withdrawn and only imipenem was continued. The infection was controlled, and the clinical symptoms and laboratory data showed obvious improvement after surgical debridement. The temperature and blood sugar level returned to normal. Two weeks after operation, total enteral nutrition was achieved. The tracheostomy tube was removed 3 weeks after the abdominal operation.

**Figure 1:**
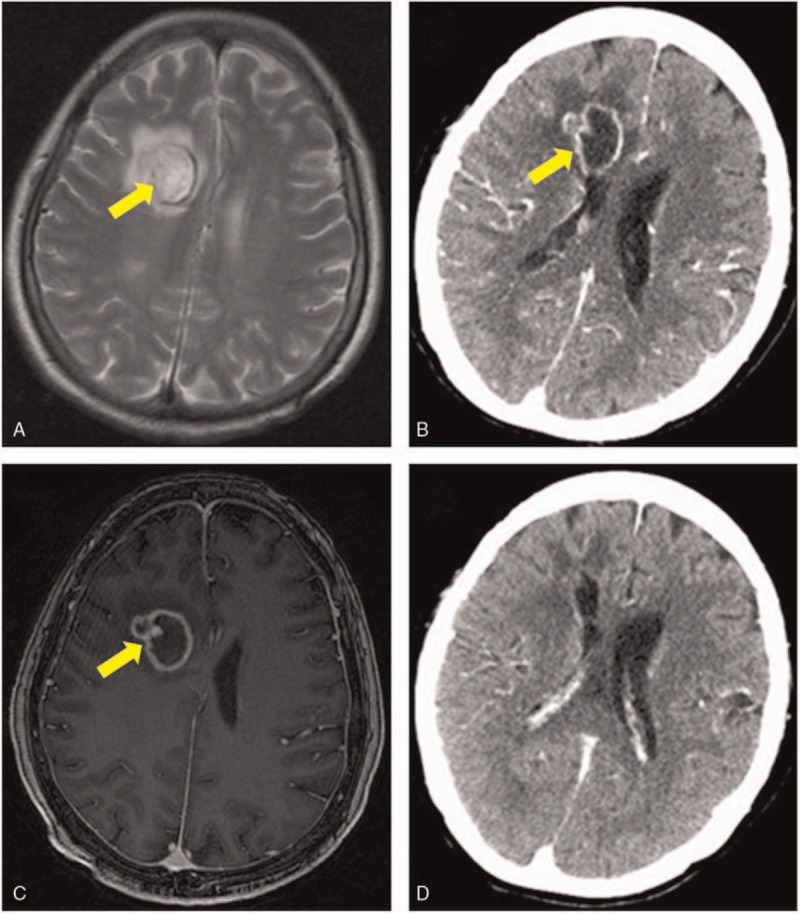
Contrast-enhanced computed tomography (CECT) and magnetic resonance imaging (MRI) of the brain lesion: (A) On day 59, a single mass is seen, with a thin, slightly irregular, hypointense ring on T2-weighted MR images (arrow marked). (B) On day 63, CECT scan shows a ring-enhancing lesion with perifocal edema in the right frontal lobe (arrow marked) (C) On day 64, an isolated abscess in the frontal lobe, with edge enhancement on contrast-enhanced MRI (arrow marked). (D) On day 87 (3 weeks after head surgery), the brain lesion has almost disappeared on CECT. CECT = contrast-enhanced computed tomography, MRI = magnetic resonance imaging

**Figure 2:**
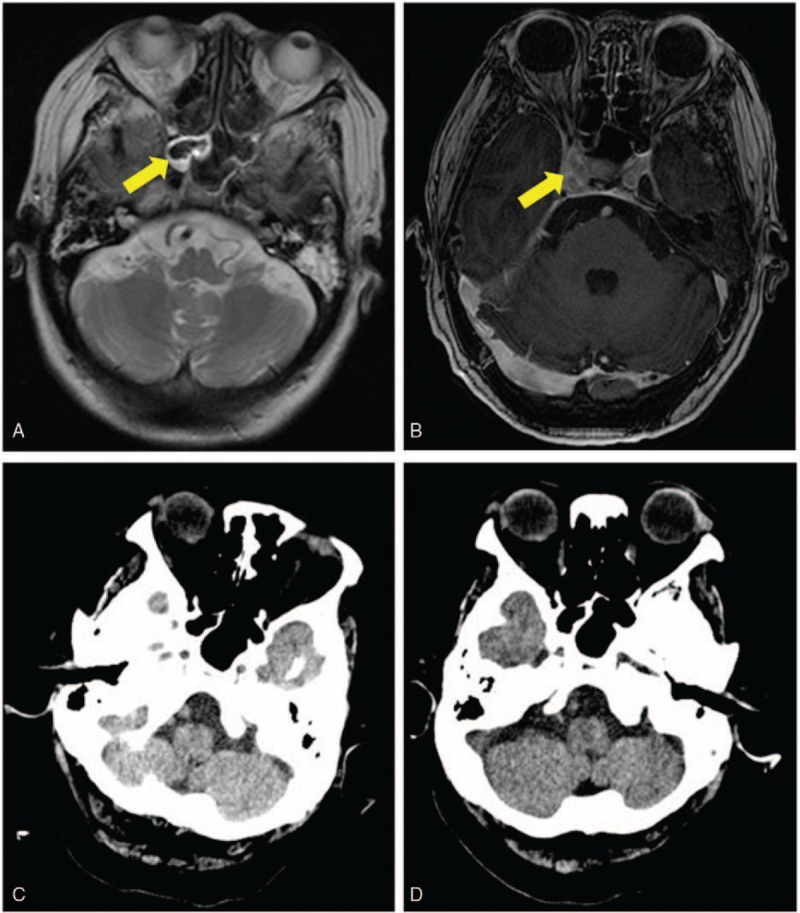
Sphenoid sinus display in MRI and CT: (A) On day 59, the right sphenoid sinus wall shows hyperintense signal on T2-weighted MR images (arrow marked). (B) On day 64, contrast-enhanced MR images shows abnormally enhancing soft tissue in the right sphenoid sinus (arrow marked). CECT on day 63 (C) and CT on day 76 (D) does not reveal the sphenoid sinus disease. CECT = contrast-enhanced computed tomography, CT = computed tomography, MRI = magnetic resonance imaging.

Four weeks after the abdominal surgery, however, the patient suddenly developed chills and fever, altered mental status, and seizures. Magnetic resonance imaging (MRI) of the head revealed a solitary 3-cm size oval mass in the right frontal lobe (Fig. 1A), with the right sphenoid sinus wall showing hyperintense signal on T2-weighted MR images (Fig. 2A). CECT of the head done 4 days later showed a ring-enhancing lesion with perifocal edema in the right frontal lobe (Fig. 1B). Contrast-enhanced MRI confirmed the right sphenoid sinus disease (Fig. 2B) and the homolateral frontal lobe mass with edge enhancement (Fig. 1C). Infection workup confirmed the diagnosis of cerebral abscess, and neuronavigation-guided aspiration and drainage was performed. Cytopathology and culture of surgically aspirated specimens revealed hyphae and confirmed that the brain abscess was due to Aspergillus. The serum was also positive for galactomannan antigen. CT scan and respiratory culture ruled out invasive pulmonary aspergillosis. Intravenous voriconazole (6 mg/kg twice a day on day 1, followed by 4 mg/kg twice daily) was initiated as soon as the diagnosis was established. After 4 weeks of treatment with voriconazole, her symptoms were improved and the brain lesion had almost disappeared on CECT (Fig. 1D). Then, she was discharged without complications.

After discharge, the patient could not afford to continue voriconazole therapy and did not take any other antifungal treatment; she refused further intervention, and died 6 months after discharge.

## 3 Discussion

Aspergillus is a ubiquitous saprophytic fungus found in soil, growing as a mold on decaying vegetable matter. Aspergillus fungal spores can be inhaled into the upper respiratory tract and survive as a commensal of the paranasal sinuses; it can also colonize the external auditory canal.^[5]^ Depending on the host's immune status, a variety of diseases can be caused by Aspergillus spp., ranging from allergies and superficial infections to life-threatening invasive mycoses. Inhalation of Aspergillus spp. conidia by immunocompetent individuals rarely causes disease as they are efficiently eliminated by immune mechanisms.^[6]^ However, individuals with compromised immune systems are at high risk of developing a fatal fungal infection. The continuing advances in medical care, with many patients surviving with chemotherapy and immunosuppressive therapies (following transplants), has contributed to the steadily increasing number of patients with impaired immune systems.^[7]^ Rarely, infection may occur in apparently immunocompetent patients. Our patient, for instance, can be considered to have been immunocompetent. The well-encapsulated lesion seen on her MRI and CECT was evidence of competent host defense mechanisms attempting to isolate or encapsulate the infecting organism. However, the course of severe acute pancreatitis and the extended stay in the ICU, with multiple invasive procedures, poorly controlled hyperglycemia, sepsis-associated immunoparalysis, prolonged antibiotic and intermittently corticosteroid use, all contributed to the development of invasive aspergillosis (IA) in our patient. Existing researches show that extended ICU stay, hemodialysis, advanced liver disease, antibiotics, low-dose steroids, congestive heart failure, chronic obstructive pulmonary disease, mechanical ventilation, and diabetes are known risk factors for IA in ICU patients without classical immunosuppression.^[4,8]^

Severe acute pancreatitis results in marked metabolic stress, and causes a state of hyperglycemia. Alterations in glucose and insulin regulation adversely affect the function of the cellular components of the innate immune system.^[9]^ In combination with inadequate systemic insulin levels and insulin resistance, long-term exposure to stress-induced hyperglycemia is linked to increased incidence of infections and sepsis, multiorgan failure, and mortality.^[10]^ During the course of INP, sepsis-associated immunoregulatory disturbances, such as macrophage deactivation and altered cellular immune response, can induce a state of immunoparalysis, hampering host response to fungal infection.^[11]^ Treatment with broad-spectrum antibiotics is at the cost of increased risk of infection with multidrug-resistant bacteria or opportunistic microorganisms. Administration of broad-spectrum antibiotics over a 3-month period has been shown to predispose to central nervous system aspergillosis.^[12]^ Our patient had been on broad-spectrum antibiotics for 8 weeks, consequently, developed multidrug-resistant *A baumannii* infection and concomitantly with CA. The elevated blood sugar also likely promoted the development CA.^[13]^ A high prevalence of diabetes mellitus has reported in immune-competent patients with CA.^[14]^ Diabetes is known to have an immunosuppressive effect, but the relationship between persistent hyperglycemia and CA has not been studied. In our case, depressed phagocyte activity induced by persistent hyperglycemia and insulin resistance might promote survival of Aspergillus in mucosal surfaces through a decrease in mucosal clearance of the fungus.

Aspergillus can reach the brain by 3 different routes: by hematogenous spread from a remote extracranial focus; by extension from a contiguous extracranial location; and by direct introduction consequent to mechanical breakdown of the blood–brain barrier due to surgery or trauma.^[15]^ Our patient had not undergone any neurosurgical procedure previously and had no evidence of invasive pulmonary aspergillosis. She did, however, have homolateral sinus disease, and the location of the cerebral abscess suggested that the source of infection may have been a small focus in the paranasal sinuses, with infection reaching the brain via direct extension or the hematogenous route. In the literature, CT scan has been mentioned as the modality of choice for identifying paranasal sinus disease. In our patient, CT scan could not identify the focus of infection in the paranasal sinus (Fig. 2C and D), but, MRI readily revealed the tiny sinus lesions (Fig. 2A and B).

The gold standard of systemic antifungal treatment is voriconazole; it is significantly superior to amphotericin B and its use has led to a profound improvement in the survival rates of patients with CA.^[16]^ Liposomal amphotericin B appears to be an alternative for primary treatment, while caspofungin, amphotericin B lipid complex, or posaconazole are capable of inducing partial or complete response in patients refractory to, or intolerant of, primary antifungal therapy. Generally, antifungal therapy should be continued until the manifestations of IA have been completely resolved or until residual scarring is demonstrated with imaging, which may take up to 12 weeks. Our patient only administered with voricanazole antifungal therapy for 4 weeks, and failed to continue antifungal therapy maybe give rise to her death 6 months later.

Surgical drainage or excision, tailored to the location and size of the lesion, is an important adjunct to antifungal therapy and may be life saving. Surgery can decrease the fungal load, allow better antifungal penetration, relieve mass effect, and decrease the local neurotoxic and inflammatory effects of the fungal infection.^[17]^ Neurosurgical intervention combined with antifungal therapy has provided better survival rates than pharmacologic treatment alone.^[2,18,19]^

In conclusion, CA may occur in immunocompetent patients. MRI precedes CT on identify the latent sinus disease—the possible source of the Aspergillus infection. Persistent hyperglycemia, sepsis-associated immunoparalysis, and prolonged antibiotic use could impair severe patient's immunocompetence, making them more susceptible to opportunistic cerebral Aspergillus infection; the risk may be especially high in patients with paranasal sinus diseases. The combination of timely neurosurgical intervention and voriconazole antifungal therapy appears to have a favorable outcome.
